# Incidental Finding of Hepatic Inflammatory Pseudotumor Immunoglobulin G4-Related Disease With Underlying Chronic Hepatitis C

**DOI:** 10.7759/cureus.44066

**Published:** 2023-08-24

**Authors:** Malique Delbrune, Nicha Wongjarupong, Elizabeth S Aby, Carlos Iwamoto, Mohamed Hassan

**Affiliations:** 1 Gastroenterology, Hepatology and Nutrition, University of Minnesota, Minneapolis, USA; 2 Gastroenterology, Hepatology and Nutrition/ Transplant Hepatology, University of Minnesota, Minneapolis, USA; 3 Laboratory Medicine and Pathology, University of Minnesota, Minneapolis, USA

**Keywords:** case report, gastroenterology, igg4 -related disease, hepatic pseudotumor, chronic hepatitis c virus

## Abstract

Immunoglobulin G4-related disease (IgG4-RD) is a unique immunological disease that can impact multiple organs including a formation of a hepatic inflammatory pseudotumor (IPT). We present a case of a 67-year-old male with a history of chronic viral hepatitis C infection who had an accidental finding on magnetic resonance imaging (MRI) of a liver arterially enhancing lesion. With an extensive work-up, immunohistochemical stains for immunoglobulin G of the liver lesion was performed and showed markedly increased IgG4-positive plasma cells (> 50/HPF), which was consistent with hepatic inflammatory pseudotumor related to IgG4-RD. The patient was treated with prednisone with a complete resolution of the hepatic lesion. The diagnosis of hepatic IPT and IgG4-RD requires a high degree of clinical suspicion and coordination with a multi-disciplinary team, including pathologists. Early tissue acquisition and staining for IgG4 was essential for the early diagnosis and treatment in this case. We also provide a comprehensive summary of published reports of IgG4-RD presenting with IPT.

## Introduction

Immunoglobulin G4-related disease (IgG4-RD) is an immune mediated condition that can affect multiple organs and lead to organ failure [1]. The presentation of IgG4-RD can vary from asymptomatic to subacute multi organ failure; on occasion patients can present with mass-like lesions, which can be concerning for malignancy [1]. IgG4 related inflammatory pseudotumor (IPT) presenting in the liver is a rare manifestation of IgG4-RD with few cases described [2]. Hepatic IPTs make up roughly 8% of lesions associated with IgG4-RD [3]. We present a patient with history of laparoscopic cholecystectomy complicated by common hepatic artery transection and damage to the confluence of right anterior and left hepatic ducts and chronic hepatitis C with incidental finding of hepatic IPT confirmed on liver biopsy.

## Case presentation

**Figure 1:**
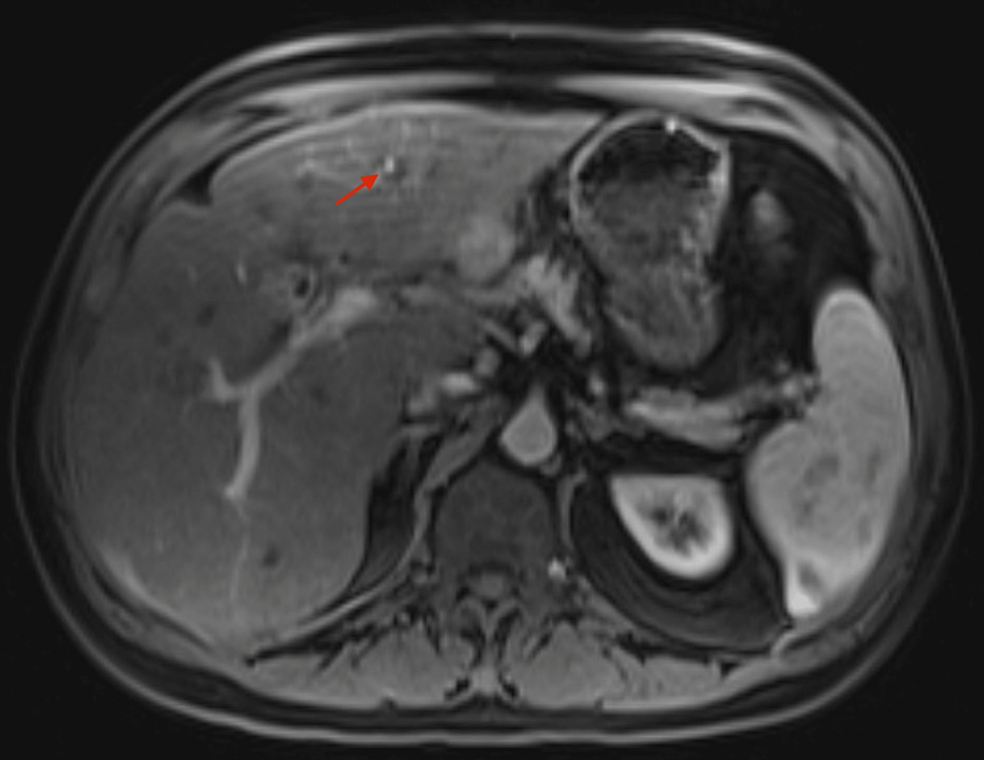
MRI image Magnetic resonance imaging (MRI) revealed an arterially enhancing lesion arising exophytically in segment III of the liver with associated washout and pseudocapsule (red arrow).

A 67-year-old male with a history of chronic viral hepatitis C (with a sustained virologic response following treatment with velpatasvir-sofobuvir and ribavirin seven years prior) without advanced hepatic fibrosis presented with a bile duct injury following laparoscopic cholecystectomy for symptomatic acute cholecystitis. His post operative course was complicated by multiple instances in which percutaneous drains placed at the time of surgery were dislodged and during one drain replacement, the tube was passed through the hepatic venous system and pleural space with subsequent bilo-thorax development. Magnetic resonance imaging (MRI) for drain assessment found an enlarging arterially enhancing lesion involving hepatic segment 3, measuring 2 cm, with associated washout and pseudo capsule, concerning for hepatocellular carcinoma (HCC) (Figure 1). His laboratory values at that time were alkaline phosphatase 263 U/L (40-129 U/L), aspartate aminotransferase (AST) 78 U/L (10-50), alanine aminotransferase (ALT) 102 U/L (10-50), total bilirubin 0.7 mg/dL (<1.2 mg/dL), direct bilirubin 0.29 mg/dL (0-0.30 mg/dL), international normalized ratio (INR) 1.28 (0.85-1.15), and serum creatinine 1.39 mg/dL (0.67-1.17 mg/dL).

A core needle biopsy of the liver lesion was performed and showed an inflammatory process composed of abundant lymphoplasmacytic inflammation with focal storiform-type fibrosis. Immunohistochemical stains for Immunoglobulin G (IgG) performed showed markedly increased IgG4 positive plasma cells (> 50/HPF), which was consistent with IgG4-RD. Alpha-fetoprotein collected two months prior was 4 ng/mL (<8.3 ng/mL). Immunological staining of the patient’s gallbladder from his prior cholecystectomy did not demonstrate an elevated IgG4 to IgG plasma cell ratio suggesting IgG4-RD in the gallbladder.

**Figure 2:**
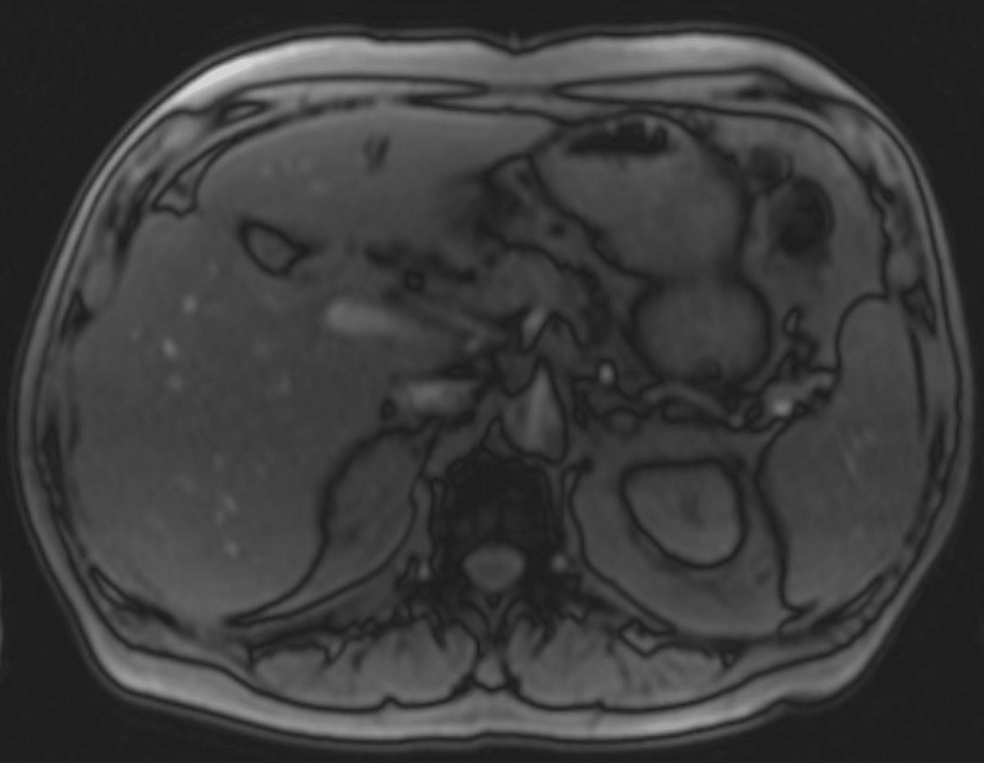
Follow-up MRCP imaging Magnetic resonance cholangiopancreatography (MRCP) image demonstrating lack of visualization of a previously noted lesion in segment III of the liver.

The patient was started on a prednisone taper plan as follows: 40 mg for 14 days, then 20 mg for 14 days, and then 10 mg for 14 days. Following the first two-week course of steroid treatment, his laboratory studies were as follows: alkaline phosphatase 125 U/L (40-126 U/L), AST 38 U/L (10-50 U/L), ALT 57 U/L (10-50 U/L), total bilirubin 0.5 mg/dL (<1.2mg/dL), direct bilirubin 0.28 mg/dL (0.0-0.30 mg/dL), INR 1.41 (0.85-1.15), and serum creatinine 2.56 mg/dL (0.67-1.17 mg/dL). After the patient had completed the steroid treatment plan, follow-up imaging was done. The visualized mass-like structure was not visualized on magnetic resonance cholangiopancreatography (MRCP) (Figure 2). The serum IgG4 level collected after the steroid treatment, which was three months following the diagnosis, was 34 mg/dL (4-86 mg/dL).

## Discussion

Hepatic IPT is a rare benign tumor that can mimic liver cancer, such as HCC, on imaging. When evaluating hepatic lesions, cross-sectional imaging such as MRI and computerized tomography (CT) are often unable to differentiate concerns for malignancy from IgG4-RD. In the presenting case, there was a high suspicion of HCC because the lesion was exophytic, had enhancement on imaging, and the patient had a history of hepatitis C, although the patient had no evidence of advanced fibrosis. The diagnosis of IgG4-RD can be made clinically using the Japanese Comprehensive Clinical Diagnostic (CCD) criteria for IgG4-RD and histopathology [4]. The Japanese CCD 2011 criteria for IgG4-RD are as follows: organ involvement, such as diffuse/localized swelling; elevated serum IgG4 concentrations > 135 mg/dl; and marked plasmacyte infiltration, defined as >10 IgG4+ cells per high-powered field (HPF) and a > 40% ratio of IgG4+/IgG + cells, accompanied by fibrosis on histopathological examination [4]. The criteria for IgG4-RD was updated in 2020 by the original Japanese IgG4-RD team with the following change to clinical presentation: clinical and radiological features of one or more organs showing diffuse or localized swelling or a mass or nodule characteristic of IgG4-RD and in single organ involvement, lymph node swelling is omitted [4]. Laboratory findings of IgG4 serum concentration greater than 140 mg/dL are seen in more than 70% of patients and elevation at least two times the upper limit of 140 mg/dL is 99% specific for IgG4-RD [5].

The patient did not obtain a serum IgG4 level until after steroid treatment and at that time, serum IgG4 levels were found to be within normal limits. A biopsy is essential to make the definitive diagnosis of IgG4-RD. The pathognomonic histology for IgG4-RD includes a dense lymphoplasmacytic infiltrate with a storiform pattern, evidence of obliterative phlebitis, and moderate eosinophilic infiltrate that is positive for IgG4 plasma cells; a diagnosis requires two out of three findings [1,6,7]. The patient’s liver biopsy met these diagnostic criteria. There is a concern for seeding of the malignancy during a biopsy; seeding has been seen in 2.7% of cases following the biopsy of a malignant liver tumor [8]. If the etiology of the mass is unclear following biopsy and there is a high suspicion of malignancy, surgical resection can be performed. Treatment for IgG4-RD relies on steroids or other immunosuppressants. The current recommendation for treatment includes four weeks of prednisone at a dose of 30-40 mg daily with a taper every two weeks of 5 mg or 0.6 mg/kg every 2-4 weeks over 3-6 months [1,9]. Some cases have been shown to resolve spontaneously without steroid treatment at 1-year follow-up [3,10].

A literature search via PubMed using key terms -IgG4, -hepatic, and -pseudotumor found 38 case reports. After excluding cases without access to full text (n= 18) and patients of <18 years old (n= 1), 19 case reports remained (Table 1). The mean age of presentation was 60.7 years. The majority of Ig4-related liver diseases occurred in males (84%). A common factor amongst cases was that imaging findings were inconclusive in determining if the etiology was malignant or infectious. Ten of the 19 cases reviewed provided serum IgG4 levels, 70% (7/10) of the cases did not meet the serum IgG4 levels proposed by Nambiar and Oliver [5], which were proposed to be 99% specific for the diagnosis of IgG4-RD. Of all the papers found, there were no other case reports involving a patient with hepatic pseudotumor from IgG4-RD and a history of hepatitis C. The association between hepatitis C and IgG4-RD is currently unknown. One potential mechanism for the relationship is that immunoglobulins, such as IgG4, play a role in the disease course of both conditions. One study proposed that in hepatitis C infections, the presence of IgG4 antibodies was correlated with lower levels of viremia [11]. However, further study is needed to determine the link between IgG4 levels, IgG4-RD and hepatitis C.

**Table 1:** Review of published case reports of hepatic pseudotumor IgG4-RD.

Article	Age	Sex	Tumor Location	Biopsy	Biopsy Method	Resection	Initial Diagnostic Impression	Serum IgG4	Imaging	Diagnosis Criteria	Previous malignancy	Treatment	History of Hepatitis C
Jandee and Boonsrib, 2020 [12]	50	Male	Hepatic segment 5	Yes	Ultrasound-guided liver biopsy	No	Parasitic infection,chronic abscess	5330 mg/dL	Abdominal CT, MRI	Serum immunoglobulin	No	Steroid (prednisolone 40mg/day) Azathioprine 50mg/day for maintenance after 3 months of starting steroid	No
Shibata et al., 2016 [8]	72	Male	Hepatic segment 7	Yes	Ultrasound using 18 gauge needle	No		137 mg/dL	CT		Yes, Hilar cholangiocarcinoma and bile duct carcinoma	Antibiotics initially due to diabetes, nsaids and ursodeoxycholic acid. Symptoms persisted so steroids started	no
Legkiy et al., 2017 [13]	60	Male	Hepatic segments 2 and 6	Yes	Ultrasound guided percutaneous needle biopsy	No		Did not quantify	CT	IgG4 levels serum of 19.1g/l			No
Yoon et al., 2022 [9]	59	Male	Hepatic hilum unspecified segment	Yes	CT-guided biopsy	No		354 mg/dL		Elevated IgG4, histology	No	Prednisone	no
Miyagi et al., 2022 [3]	66	Male	Hepatic segment 4	Yes	ultrasonography-guided puncture	No		Elevated, specific value not given	CT imaging	Immunoglobulin level	No	No antibiotics after MRCP draining stones in bile duct, inflammation improved	No
Hamano et al., 2021 [6]	71	Male	Hepatic segment 3	Yes	Ultrasound Guigui biopsy	No	Intrahepatic cholangiocarcinoma	205 mg/DL (5-117 mg/dL)	Abdominal CT initially, MMRI		IgG4 RD Renal disease	10mg prednisone daily	No
Naitoh et al., 2009 [14]	77	Male	Hepatic segment 3	No	No	Yes, left lobectomy	Intrahepatic cholangiocarcinoma	231 mg/DL (normal <134 mg/dL)	CT imaging, MRI, MRCP, ERCP		No	Surgical Resection	No
Kaneko et al., 2017 [15]	73	Male	Left lobe of liver mass unspecified segment	Yes	Ultrasound guided biopsy	No	Hepatic IPT, DLBCL	Elevated, specific value not given	CT imaging		Pancreatic head mass cancer	R-CHOP chemotherapy for Diffuse large b-cell lymphoma on liver,	No
Lee et al., 2018 [2]	67	Male	Right hepatic lobe unspecified segment	NO	No	Yes,Resection of right lower lobe	Sarcomatous intrahepatic cholangiocarcinoma	Did not assess before removal	CT imaging, MRI			Surgical resection done	No
Kim et al., 2011 [16]	58	Male	Along the left portal vein unspecified	Yes	Percutaneous biopsy		IgG4 and malignant lymphoma	Elevated, specific value not given	CT imaging	Biopsy showing elevated IgG4 staining	No	30 mg prednisone	No
Patel et al., 2018 [10]	48	Male	Right lobe of liver unspecified	Yes	Did not specify	No	Chronic pancreatitis causing inflammation	114 mg/dL	CT imaging, MRI	Pathology	None	None	No
Primitivo et al., 2021 [17]	62	Female	Central liver mass	Yes	Percutaneous needle	No	Did not state	non elevated 72.5 mg/dL	Ultrasound, CT	Pathology showing active cholangitis with lymphoplasmacytic infiltration and fibrosis	None, HIV positive	glucocorticoids 40mg	No
Koiwai et al., 2021 [18]	71	Male	Liver hilum	Yes	Ultrasound guided fine needle biopsy	No	Did not state	465 mg/dL	CT imaging, MRCP		Pancreatic head tumor	Prednisone 0.6mg/kg	No
Miyajima et al., 2017 [19]	50	Female	Lateral lobe unspecified	Yes	Unspecified	No	Cholangiocellular carcinoma, hepatocellular carcinoma, metastatic liver tumor, actinomycosis	241 mg/dL	Ultrasound, CT	Elevated serum IgG4, random samplings showing elevated plasma cells	No	Antibiotics initially, prednisolone 0.5mg/kg	No, Hepatitis C antibodies negative
Kanno et al., 2005 [20]	48	Male	Left hepatic lobe unspecified segment	No	No	Left lobectomy	Autoimmune pancreatitis with multiple metastatic liver tumors from cholangiocellular carcinoma		Ultrasound, CT, ERCP	Histology showing diffuse and dense lymphoplasmacytic infiltration with obliterating phlebitis	None	Corticosteroid treatment	No
Mulki et al., 2015 [7]	50	Male	Right lobe of liver unspecified segment	Yes	Ultrasound guided	No	Multifocal necrotic hepatic abscesses with septic thrombus, possible malignancy		Ultrasound, CT		No	Antibiotics initially, did not start immunotherapy because patient was asymptomatic	No
Uchida et al., 2007 [21]	54	Male	Hepatic segment IV	Yes	Did not state	No	Pancreatic cancer with metastasis		Ultrasound, CT	Biopsy pathology	No	None, resolved on its own, patient was asymptomatic	No, Hepatitis C antibodies negative
Okamura et al., 2021 [22]	67	Male	Left umbilical tumor	No	did not biopsy	Yes	Intrahepatic cholangiocarcinoma	206 mg/dL	MRI	Histology	IgG4 Sclerosing cholangitis		No
Agaimy and Märkl, 2013 [23]	51	Female	Hepatic segments II/IV	No	No	Yes	Infectious etiology, AML	No serum IgG4 level	CT, Ultrasound	Histology status post resection		Surgical removal, symptoms resolved so no other intervention	No

## Conclusions

We incidentally discovered a patient with IgG4-RD with the presentation of hepatic IPT. The diagnosis of hepatic IPT and IgG4-RD requires a high degree of clinical suspicion and coordination with a multi-disciplinary team, including pathologists. The Japanese CCD criteria can be used to determine the probability of the diagnosis. In cases such as this one, serum IgG4 levels may not be drawn prior to treatment. However, it is reassuring to see normal serum IgG4 levels following steroid treatment. Early tissue acquisition and staining for IgG4 was essential for the early diagnosis and treatment in this case. Comparing this patient’s case with previously published case reports of hepatic IPT, the patient’s chronic hepatitis C was found to be unique. The exact mechanism connecting hepatitis C and IgG4-RD is still unknown requiring further study with levels of IgG4 immunoglobulin influencing each disease's progression. Our patient completed steroid treatment and follow-up imaging did not visualize the previously demonstrated mass.

## References

[1] Sharzehi K (2021). Hepatic manifestations of immunoglobulin G4-related disease. Clin Liver Dis (Hoboken).

[2] Lee JH, Kim HS, Kim JS, Lee DK, Lim JH (2018). Hepatic actinomycosis with immunoglobulin G4-related liver disease: a case report. Medicine (Baltimore).

[3] Miyagi A, Fujimoto D, Yoshikawa A (2022). A rare case of fibrohistiocytic hepatic inflammatory pseudotumor with cholecystocholangitis showing positive IgG4 staining. Clin J Gastroenterol.

[4] Umehara H, Okazaki K, Kawa S (2021). The 2020 revised comprehensive diagnostic (RCD) criteria for IgG4-RD. Mod Rheumatol.

[5] Nambiar S, Oliver T (22). IgG4 Related Disease. http://www.ncbi.nlm.nih.gov/books/NBK499825/.

[6] Hamano A, Yamada R, Kurata K (2021). Difficulty in differentiating between IgG4-related hepatic inflammatory pseudotumor and intrahepatic cholangiocarcinoma. Clin J Gastroenterol.

[7] Mulki R, Garg S, Manatsathit W, Miick R (2015). IgG4-related inflammatory pseudotumour mimicking a hepatic abscess impending rupture. BMJ Case Rep.

[8] Shibata M, Matsubayashi H, Aramaki T, Uesaka K, Tsutsumi N, Sasaki K, Ono H (2016). A case of IgG4-related hepatic inflammatory pseudotumor replaced by an abscess after steroid treatment. BMC Gastroenterol.

[9] Yoon J, Hu S, Phillips D, Fathi A, Ameer A (2022). IgG4-related hepatic pseudotumor masquerading as a Klatskin tumor. Case Reports Hepatol.

[10] Patel H, Nanavati S, Ha J, Shah A, Baddoura W (2018). Spontaneous resolution of IgG4-related hepatic inflammatory pseudotumor mimicking malignancy. Case Rep Gastroenterol.

[11] Santos VC, Schinoni MI, Oliveira IS, Atta ML, Atta AM (2019). IgG1 and IgG4 antibodies against Core and NS3 antigens of hepatitis C virus. Rev Soc Bras Med Trop.

[12] Jandee S, Boonsri P (2020). Atypical manifestations of IgG4-related disease as multiple liver abscesses with subcapsular tracts and migratory pulmonary nodules mimicking parasitic infection. Case Rep Gastroenterol.

[13] Legkiy O, Wajda J, Ćwierz A, Wysocka J, Komorowski AL (2019). Hepatic inflammatory pseudotumor related with IgG4. Gastroenterol Hepatol.

[14] Naitoh I, Nakazawa T, Ohara H (2009). IgG4-related hepatic inflammatory pseudotumor with sclerosing cholangitis: a case report and review of the literature. Cases J.

[15] Kaneko R, Mitomi H, Nakazaki N, Yano Y, Ogawa M, Sato Y (2017). Primary hepatic lymphoma complicated by a hepatic inflammatory pseudotumor and tumor-forming pancreatitis. J Gastrointestin Liver Dis.

[16] Kim F, Yamada K, Inoue D (2011). IgG4-related tubulointerstitial nephritis and hepatic inflammatory pseudotumor without hypocomplementemia. Intern Med.

[17] Primitivo A, Oliveira MH, Gonçalves A (2021). IgG4-related hepatic inflammatory pseudotumour: could MRI suggest the correct diagnosis?. BMJ Case Rep.

[18] Koiwai A, Hirota M, Satoh M (2021). Immunoglobulin G4-related hepatic inflammatory pseudotumor diagnosed with endoscopic ultrasound-guided fine-needle biopsy. Case Rep Gastroenterol.

[19] Miyajima S, Okano A, Ohana M (2017). Immunoglobulin G4-related hepatic inflammatory pseudotumor invading the abdominal wall. Clin J Gastroenterol.

[20] Kanno A, Satoh K, Kimura K (2005). Autoimmune pancreatitis with hepatic inflammatory pseudotumor. Pancreas.

[21] Uchida K, Satoi S, Miyoshi H (2007). Inflammatory pseudotumors of the pancreas and liver with infiltration of IgG4-positive plasma cells. Intern Med.

[22] Okamura Y, Nishitai R, Sasaki N (2021). Intrahepatic bile duct rupture associated with IgG4-related sclerosing cholangitis presenting hepatic inflammatory pseudotumor. Clin J Gastroenterol.

[23] Agaimy A, Märkl B (2013). Inflammatory angiomyolipoma of the liver: an unusual case suggesting relationship to IgG4-related pseudotumor. Int J Clin Exp Pathol.

